# Does it fit in your pocket? economic burden of PD-1 inhibitors' toxicity in the supplementary health system: evidence from Brazil

**DOI:** 10.1186/s12913-023-09736-6

**Published:** 2023-07-21

**Authors:** Hugo Santos Duarte, Cassia Rita Pereira da Veiga, Claudimar Pereira da Veiga, Alberto Julius Alves Wainstein, Wesley Vieira da Silva, Ana Paula Drummond-Lage

**Affiliations:** 1 Faculdade de Ciências Médicas de Minas Gerais, Alameda Ezequiel Dias, 275, Belo Horizonte, MG 30130-110 Brazil; 2 Departamento de Gestão de Serviços de Saúde, Escola de Enfermagem, Universidade Federal de Minas Gerais, Av. Alfredo Balena, 190, Belo Horizonte-MG, 30130-100 Brazil; 3 FDC Business School, Fundação Dom Cabral, , Av. Princesa Diana, 760,Alphaville Lagoa Dos Ingleses, Nova Lima, MG 34018-006 Brazil; 4 Universidade Federal de Alagoas - UFAL, Av. Lourival Melo Mota, S/N, Tabuleiro Do Martins, Maceió, Alagoas 57072-900 Brazil

**Keywords:** Health management, Burden of toxicity, Cost, Oncology, PD-1/PD-L1 inhibitor

## Abstract

**Background:**

A full understanding of the economic burden associated with treatment-related adverse events (AEs) can aid estimates of the incremental costs associated with incorporating new technologies and support cost-effective economic modeling in Brazil. In this context, the main objective of this work was to evaluate in a real-life database: (i) the direct medical cost of monitoring the occurrence of AEs (CMO); (ii) the direct medical cost of managing an identified AE (CMN); and (iii) the total direct medical cost of monitoring and managing AEs (TMC), in quarterly periods from 0 to 24 months of the monitoring of cancer patients who used a PD-1 inhibitor from the perspective of the supplementary health system in Brazil.

**Methods:**

This study was conducted from the supplementary health system (SSS) perspective and followed the methodological guidelines related to cost-of-illness studies. A bottom-up (person-based) approach was used to assess the use of health resources to monitor and manage AEs during the use of PD-1 inhibitors, which made it possible to capture differences in the mean frequency of the use of health services with stratification results for different subgroups. As the Brazilian SSS is complex, asymmetric, and fragmented, this study used information from different sources. The methodology was divided into three parts: (i) Data Source: clinical management of AEs; (ii) Microcosting: management of the economic burden of AEs; (iii) Statistical analysis: stratification of results for different subgroups.

**Results:**

Analysis of the economic burden of toxicity showed higher CMO costs than CMN in all the periods analyzed. In general, for every BRL 100 on average invested in the TMC of AEs, BRL 95 are used to monitor the occurrence of the AE and only BRL 5 to manage an identified AE. This work also showed that the sociodemographic characteristics of patients, the journey of oncological treatment, and the toxicity profile affect the economic burden related to AE.

**Conclusion:**

This study provided real-world evidence of the economic burden of AEs associated with the use of PD-1 inhibitors in Brazil. This work also made methodological contributions by evaluating the economic burden of AE of PD-1 inhibitors considering the kinetics of toxicity occurrence and categorizing the costs in terms of CMO, CMN and TMC.

**Supplementary Information:**

The online version contains supplementary material available at 10.1186/s12913-023-09736-6.

## Introduction

Immune checkpoint inhibitors are a therapeutic strategy based on applying monoclonal antibodies to block the immune escape of tumor cells [1]. The axis that involves the programmed death receptor-1 (PD-1) and its ligands (PD-L1 and PD-L2) has been consolidated as a new foundational component of therapeutic regimens across multiple tumor types in isolated use or in combination with other therapies [2]. In Brazil, the National Health Surveillance Agency (ANVISA) has approved six immune checkpoint inhibitors targeting the PD-1/PD-L1 pathway in the last five years, including three anti-PD-1 antibodies (pembrolizumab, nivolumab and cemiplimab) and three anti- PD-L1 (atezolizumab, avelumab and durvalumab), each with various therapeutic indications [3–8].

The treatment with PD-1/PD-L1 inhibitors has been a significant breakthrough in the field of oncology and represent the new standard of care for different tumor types. There are many works in the literature that show how PD-1/PD-L1 inhibitors have contributed to a significant improvement in the outcome of treatment and prognosis of cancer patients [9, 10]. On the other hand, the cost increase associated with the use of PD-1/PD-L1 inhibitors in a resource-constrained environment is a challenge for global health systems, mainly for developing countries with universal healthcare such as that offered by the Unified Health System (SUS) in Brazil [11]. With income growth and the expansion of the labor market, more people are opting for private health plans because they understand that the services provided are of higher quality than those offered by the SUS [12]. Brazil has more than 48 million beneficiaries (~ 25% of the national population) in the supplementary health system (SSS) [13], and only these beneficiaries or those patients who assume out-of-pocket costs have regular access to PD-1/PD-L1 inhibitors [11], highlighting that the emergence of new health technologies intensifies inequities in Brazilian health systems [14]. Assessing disease cost from the perspective of the SSS is fundamental for the predictability of care costs, considering that the SSS business model is based on the risk of accidents and mutualism among beneficiaries [15].

Despite important clinical benefits, cancer treatments with PD-1 / PD-L1 inhibitors are associated with a unique spectrum of treatment-related adverse events (AEs) that reflect a homeostatic imbalance in the regulation of the immune system, with multiple and not fully known pathogenesis mechanisms [16]. PD-1/PD-L1 inhibitors can trigger AEs related to immune dysregulation, which clinically manifests with symptoms similar to autoimmune diseases [9, 17, 18], as well as toxicities similar to classic cytotoxic chemotherapy [18, 19]. Despite presenting a better tolerability profile than chemotherapy [19], AEs related to PD-1/PD-L1 inhibitors have clinical manifestations, incidence and kinetics that differ from the patterns of previous treatments [16, 20], requiring new skills and knowledge for health professionals to monitor, diagnose and manage AEs. Anticipating the increasing exposure of patients to PD-1/PD-L1 inhibitor treatments, the Brazilian Society of Clinical Oncology (SBOC) organized a multidisciplinary panel that reviewed the literature and proposed general and specific guidelines related to the identification and management of AEs associated with the use of immune checkpoint inhibitors [21].

The literature on the clinical perspective of AEs associated with PD-1/PD-L1 inhibitors is vast [16–20, 22–25]. However, from an economic perspective, few scientific studies have been conducted. Therefore, most of the research that has evaluated the economic burden of the toxicity of PD-1/PD-L1 inhibitors was based on data from clinical studies [26–33] or conducted in a literature review [34, 35], and only a few studies used electronic medical records [36, 37], which are recognized as an important approach to decision making in the real world.

A full understanding of the economic burden associated with AEs can aid estimates of the incremental costs associated with adding new technologies, and support both cost-effectiveness modeling and modeling related to the assessment of the cost of disease. In this context, the main objective of this work was to evaluate the following factors in a real-life database: (i) the direct medical cost of monitoring the occurrence of AEs (CMO); (ii) the direct medical cost of managing an identified AE (CMN); and (iii) the direct medical cost for total management of an AE (TMC), composed of the sum of CMO and CMN, in quarterly periods from 0 to 24 months of the monitoring of cancer patients who used a PD-1 inhibitor in the perspective of the Brazilian SSS. In addition, this study also evaluated, as a secondary objective, the effect of three groups of variables on the three cost categories (CMO, CMN and TMC), namely: (i) the sociodemographic characteristics of patients, (ii) the PD-1 inhibitor treatment journey and (iii) the toxicity profile on the three cost categories (CMO, CMN and TMC). Previous literature shows that these variables have the potential to impact the cost of health care in the field of oncology [38–40] and therefore they will be evaluated.

## Materials and methods

**Fig. 1:**
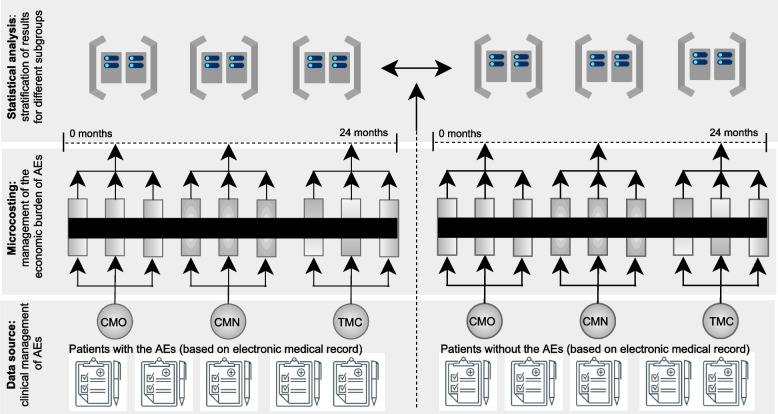
Research methodology flowchart (adapted from Larg, Moss, 2011 [41]). AE: adverse event; CMO: direct medical cost of monitoring the occurrence of AE; CMN: direct medical cost of managing an identified AE, TMC: total direct medical cost of managing AEs

This study was conducted from the SSS perspective and followed the methodological guidelines related to cost-of-illness studies [41]. A bottom-up (person-based) approach was used to assess the use of health resources to monitor and manage AEs during the use of PD-1 inhibitors, which made it possible capture differences in the mean frequency of the use of health services with stratification results for different subgroups, including those with and without AE. As CMN is an exclusive cost for patients who had AEs, it was used as a control variable to differentiate the two study populations (Fig. 1). As the Brazilian SSS is complex, asymmetric, and fragmented, this study used information from different sources. The methodology was divided into three parts, as illustrated in Fig. 1, and the source of information used in each part is shown in Table 1. More information can be found in the Supplementary Material (S1. Source of information).

**Table 1:** Source of information used to conduct each part of the research

Part of the research	Source of information
**Data Source:** clinical management of AEs	**Electronic medical records** of all patients over 18 years of age diagnosed with cancer who began treatment with a PD-1 inhibitor between January 2017 and January 2020, regardless of tumor type and with locally advanced or metastatic disease (stages III or IV)
**Microcosting:** management of the economic burden of AEs	**SBOC guidelines**: to assess recommendations on the type of health resources used to monitor and manage AEs [24]
	**Prescription information**: to define the frequency of use of health resources to monitor and to manage AEs based on the treatment dosage of the different PD-1 inhibitors [9–14]
	**Electronic medical records**: to define the real frequency of the use of health resources and the real time of drug use
	**CBHPM table**^**a**^: to evaluate costs related to medical appointments, imaging tests, laboratory tests and service rates for injectable drug treatments for outpatients [42]
	**CMED list**^**b**^: to evaluate the cost of outpatient drugs [43]
	**ANS database**^**c**^: to evaluate the cost of each inpatient procedure and the total cost of hospitalization for a given ICD [44, 45]

*AE* Adverse event, *CBHPM* Brazilian Hierarchical Classification of Medical Procedures table, *SBOC* Brazilian Society of Clinical Oncology, *CMED* Chamber of Drug Market Regulation, *ANS* National Supplementary Health Agency, *ICD* International Statistical Classification of Diseases and Related Health Problems

^a^This work used CBHPM table set 2020/2021 [42], the operating cost unit of BRL 21.89 [42] and the cost per square meter of radiological documentation of BRL 31.59 [46]

^b^This work used the Services and Merchandises Circulation Tax (ICMS) of 18% [43], and the treatment dosage of outpatient drugs followed the recommendation of the health service provider's internal guidelines and the SBOC guidelines [24]

^c^This study used the 2019 open database for Minas Gerais State [44, 45]

### Data Source: clinical management of AEs

This study used retrospective data from 2017 to 2020 obtained from electronic medical records of cancer patients treated by a unit of the largest oncology group in Brazil, located in Belo Horizonte, Minas Gerais State. Data were automatically extracted from different databases and subsequently organized in a Microsoft Excel spreadsheet based on the identification number of each patient. Records with missing information were excluded from the analysis. Three groups of data were automatically collected: (i) socio-demographic characteristics of patients (age, gender, education, marital status and previous autoimmune disease); (ii) oncological treatment journey (International Statistical Classification of Diseases and Related Health Problems [ICD], tumor type, type of PD-1 inhibitor, line of treatment, treatment regimen, length of treatment with PD-1 inhibitor, use of outpatient and inpatient resources related to AEs and reason for hospitalization); (iii) evaluation toxicity (occurrence rate, classification by anatomical site and toxicity grading). AEs were monitored at quarterly periods from 0 to 24 months of follow-up. The frequency of health resource use of outpatient and inpatient treatments related to PD-1 inhibitors’ toxicity was mapped by the electronic medical records. The research institution's information system gathers data from the anamnesis carried out in the doctor's office, the request of laboratory tests, imaging tests, cancer drugs, and supportive drugs to treat AEs. As the patient's hospitalization was carried out at a third institution different from the research institution, the inpatient resources were reported in the information system by the oncologist responsible for the patient. On the other hand, the patient's medical records did not indicate for which treatment cycle the hospital intervention was necessary. Therefore, inpatient costs were only accounted for in the total cost calculation in the 0 to 12 and 0 to 24-month intervals.

The Brazilian guideline [21] recommends that all patients receiving PD-1 inhibitors routinely undergo clinical and laboratory examination (blood count, liver, kidney, and thyroid functions) before beginning treatment and at each one or two applications, with intervals of four to six weeks during the first six months following the end of treatment. The frequency of medical appointments and laboratory tests to monitor the occurrence of AEs was based on the treatment dosage of the different PD-1 inhibitors (National Health Surveillance Agency [3–8]) and real-time drug use. In addition, as imaging tests to assess AEs are performed on demand, the real frequency of the use of this resource was validated by information from electronic medical records.

### Microcosting: management of the economic burden of AEs

To assess the economic burden related to the management of AEs, this study analyzed the direct medical costs of AEs for cancer patients who used a PD-1 inhibitor at some point in their treatment journey. The cost of health resources used by patients to manage AEs was divided and categorized into CMO, CMN, and TMC. As the cost of each resource used depends on the remuneration model and confidential commercial agreement made by the health service provider and the respective health maintenance organization (HMO) of the SSS beneficiary, this work used different sources of information to assess the direct medical cost for each resource. The Brazilian regulation of drug prices is the responsibility of the Chamber of Drug Market Regulation (CMED), and the cost of outpatient drugs was evaluated on the CMED list using the Services and Merchandises Circulation Tax (ICMS) of 18% [42]. This work also considered the service rate for injectable drug treatments for outpatients, and these costs were assessed by a standardized price list [46]. This analysis did not consider the cost of medications purchased directly by the patient, as the present study deals with the SSS perspective. The costs of laboratory tests, imaging tests, and medical consultations were also assessed by a standardized price list [46]. The cost related to the use of inpatient resources was assessed by the open data portal of the National Supplementary Health Agency [43]. More details on the composition of the cost of each health resource can be found in the Supplementary Material (S1. Source of information).

### Statistical analysis: stratification of results for different subgroups

Statistical analyses were performed using XLSTAT 2021.3.1169 software. Since the dependent variables CMO, CMN and TMC did not present normality or homogeneity of variance, the categorical groups were compared using the Mann–Whitney U or Kruskal–Wallis nonparametric tests. A bilateral alpha error of 0.05 was used to determine statistical significance.

### Compliance with ethical guidelines

The project was approved by the Research Ethics Committee of the Faculty of Medical Sciences (Faculdade Ciências Médicas) of Minas Gerais State (CAAE:67,282,617.9.0000.5134).

## Results

### Data Source 1: clinical management of AEs

The research in electronic medical records included 170 cancer patients who used a PD-1 inhibitor at some point in their treatment journey, which occurred between 2017 and 2020 with an average time of drug use of 11.0 ± 7.3 months. Of these, 66.5% were men, 53.5% were over 70 years of age at diagnosis, 65.9% were married and 73.5% had completed high school or higher education. None of the patients had a history of autoimmune disease. Given the range of therapeutic indications for PD-1 inhibitors, this study identified patients diagnosed with 15 different ICDs, of which malignant neoplasm of the bronchi and lungs was predominant (C34, 38.9%), malignant melanoma of the skin (C43, 27.1%), kidney malignancy (C64, 16.5%) and bladder malignancy (C67, 7.1%). Among the PD-1 inhibitors, the use of nivolumab was predominant (117 cases, 40.2% for C34, 28.6% for C43, 23.9% for C64 and 7.7% for other ICDs), followed by pembrolizumab (48 cases, 39.6% for C34, 27.1 for C43, 25.0% for C67 and 8.3% for other ICDs) and cemiplimab (5 cases, all classified as other ICDs). Most patients used PD-1 inhibitor monotherapy (84.1%) after the failure of previous lines of treatment (82.9%). Only 7.6% of patients used inpatient resources to manage their AEs, and 71.2% of all the medical records were of beneficiaries of a single HMO in the cooperative modality.

This study showed that 90% of the patients who used PD-1 inhibitors had some form of AE in the period of 0 to 24 months of follow-up. Based on the range of grades from 1 to 5 of Common Terminology Criteria for Adverse Events (CTCAE) (US National Cancer Institute)^1^^*^[44], 60.5% of these patients had at least one grade 2 AE and 8.8% a grade 3 AE within the 0-to-24-month follow-up period. Treatment-related toxicities occurred more frequently in the first 6 months of PD-1 inhibitor use, with 51.9% and 27.0% of patients having two distinct AEs concomitantly in periods 0 to 3 and 4 to 6 months, respectively. Grade 1 and 2 AEs were predominant, and the main anatomical sites involved in the first months of PD-1 inhibitor use were the gastrointestinal tracts and the skin. More details on the characterization of the rate of occurrence, grading and classification of treatment-related AE by the anatomical site can be found in the Supplementary Material (Tab S1).

### Microcosting: management of the economic burden of AEs

Table 2 shows the median and mean economic burden of CMO, CMN and TMC in quarterly periods from 0 to 24 months of follow-up after the beginning of treatment with a PD-1 inhibitor. In general, it can be seen that the mean CMO remains constant over time, except for the period from 0 to 3 months, in which the costs of monitoring the baseline period prior to the use of the PD-1 inhibitor were absorbed. CMN is a cost exclusively for patients who had AEs and, therefore, it is the cost that differentiates the two study populations. CMN was lower than CMO during all the periods in question.

**Table 2:** Characterization of the economic burden of treatment-related adverse events (AEs) in quarterly periods from 0 to 24 months of follow-up

**Period (months)**		**CMO (BRL)**		**CMN (BRL)**		**TMC (BRL)**	
**0 to 3**	*N*	Median	Mean ± SD	Median	Mean ± SD	Median	Mean ± SD
Total	170	4,346	4,060 ± 627	194	398 ± 2,778	4,346	4,458 ± 2,867
With AEs	102	4,346	4,078 ± 706	225	664 ± 3,569	4,571	4,742 ± 3,660^*^
Without AEs	68	4,346	4,032 ± 490	0	0	4,346	4,032 ± 490^*^
**4 to 6**							
Total	134	3,201	2,884 ± 630	0	442 ± 3,128	3,201	3,326 ± 3,217
With AEs	74	3,201	2,908 ± 715	225	800 ± 4,188	3,426	3,709 ± 4,279^*^
Without AEs	60	3,201	2,853 ± 509	0	0	3,201	2,853 ± 509^*^
**7 to 9**							
Total	101	3,201	2,824 ± 735	0	71 ± 116	3,201	2,895 ± 774
With AEs	34	3,201	3,149 ± 821	225	210 ± 104	3,429	3,359 ± 824^*^
Without AEs	67	3,201	2,660 ± 633	0	0	3,201	2,660 ± 633^*^
**10 to 12**							
Total	82	3,201	2,726 ± 622	0	53 ± 110	3,201	2,779 ± 610
With AEs	20	2,134	2,610 ± 545	225	219 ± 115	2,553	2,833 ± 496
Without AEs	62	3,201	2,762 ± 645	0	0	3,201	2,762 ± 645
**13 to 15**							
Total	65	3,201	2,736 ± 686	0	40 ± 105	3,201	2,777 ± 695
With AEs	10	3,201	2,794 ± 762	225	262 ± 119	3,426	3,056 ± 757^*^
Without AEs	55	3,201	2,726 ± 678	0	0	3,201	2,726 ± 678^*^
**16 to 18**							
Total	47	3,201	2,838 ± 499	0	27 ± 82	3,201	2,865 ± 517
With AEs	5	3,201	3,094 ± 238	225	255 ± 67	3,426	3,349 ± 172^*^
Without AEs	42	3,201	2,807 ± 514	0	0	3,201	2,807 ± 514^*^
**19 to 21**							
Total	32	3,201	2,751 ± 509	0	12 ± 47	3,201	2,763 ± 496
With AEs	2	2,134	2,134 ± 0	188	188 ± 53	2,322	2,322 ± 53
Without AEs	30	3,201	2,792 ± 499	0	0	3,201	2,792 ± 499
**22 to 24**							
Total	21	3,201	2,845 ± 568	0	0	3,201	2,792 ± 499
With AEs	0	0	0	0	0	0	0
Without AEs	21	3,201	2,845 ± 568	0	0	3,201	2,792 ± 499
**0 to 12**^‡^							
Total	170	9,682	9,326 ± 3,861	409	1,199 ± 4,158	10,091	10,525 ± 5,414
**0 to 24**^‡^							
Total	170	9,682	12,026 ± 7,326	418	1,224 ± 4,157	11,045	13,250 ± 8,051

*AE* Treatment-related adverse event, *SD* Standard deviation, *CMO* Direct medical cost of monitoring the occurrence of AEs, *CMN* Direct medical cost of managing an identified AE, *TMC* Total direct medical cost of managing AEs

^‡^Includes cost related to the use of inpatient resources. The mean difference among the groups is statistically significant at the 0.05 level (^*^p < 0.05)

The TMC results are directly influenced by the CMN and significantly higher in different analysis periods for cancer patients who had AEs (highlighted in Table 2). As the costs related to using inpatient resources were considered only for the periods of 0 to 12 and 0 to 24 months of follow-up, the economic burden was also evaluated in these specific periods. The results showed that the AE occurrence rate had an effect on CMN, CMO and TMC in the period of 0 to 24 months of follow up, with higher costs for cancer patients who had AEs and who used inpatient resources during that period.

### Stratification of results for different subgroups

Evaluation of CMO, CMN and TMC according to the sociodemographic characteristics of cancer patients.

Overall, the results show a trend that the age of the cancer patient has an impact on CMO and TMC. Patients under 65 years of age tended to have lower CMO and TMC. On the other hand, CMN is impacted by the level of education of cancer patients. Patients with complete higher education tended to have higher CMN than patients who had completed secondary education. The result of paired multiple comparisons for the gender and marital status of the cancer patient showed no significant difference in CMO, CMN and TMC during the periods in question. The mean cost of CMO, CMN and TMC based on the sociodemographic characteristics of cancer patients is shown in Table 2S.

Evaluation of CMO, CMN and TMC according to the treatment journey of cancer patients.

The results of this work showed that the tumor type impacts CMO, CMN and TMC in some follow-up periods. The ICD 10 C64 is the tumor type with the highest CMO and TMC, while the ICD 10 C67 had the lowest CMO and TMC compared with the other tumor types. The mean cost of CMO, CMN and TMC based on the tumor type of cancer patients is shown in Table 3S. The type of PD-1 inhibitor used by cancer patients also has an impact on CMO and on TMC but not on CMN. Overall, this analysis showed that nivolumab has higher CMO and TMC than the other PD-1 inhibitors. There is no significant difference between pembrolizumab and cemiplimab. The mean cost of CMO, CMN and TMC based on the PD-1 inhibitors used by cancer patients is shown in Table 4S.

Likewise, the line of treatment of cancer patients tends to have impact on CMO, CMN and TMC. Overall, CMN and TMC tend to be higher for patients who underwent palliative systemic treatment before using the PD-1 inhibitor, and CMO tends to be higher for patients who used the PD-1 inhibitor as first-line treatment. The results of this study also demonstrated that the time of exposure to PD-1 inhibitor treatment affects CMO, CMN and TMC. In general, CMO increased with the time of PD-1 inhibitor use, while CMN showed a downward trend over the period, with a higher cost in the first quarter. TMC, in turn, also showed increasing values with the time of use of the PD-1 inhibitor, mainly driven by CMO values. The mean cost of CMO, CMN and TMC based on the line of treatment and time of exposure to the PD-1 inhibitor is shown in Table 5S.

Finally, it is important to emphasize that the treatment regimen with the PD-1 inhibitor (alone or in combination) did not affect CMO, CMN and TMC in any of the periods evaluated, despite the tendency for costs to be higher with the combined treatment regimen. Conversely, the use of inpatient resources to deal with PD-1 inhibitor toxicity had an effect on CMN and on TMC. The costs of inpatient resources meant that CMN and TMC were higher for cancer patients who used this resource. The mean cost of CMO, CMN and TMC based on the treatment regimen with the PD-1 inhibitor and the use of inpatient resources is shown in Table 6S.

Assessment of CMO, CMN, and TMC for different classifications of AEs.

The mean cost of CMO, CMN, and TMC for different classifications of AEs is shown in Table 7S and Table 8S. The AE classification impacted CMO, CMN and TMC in different periods of evaluation. In general, it can be noted that the subgroups of patients with pulmonary AEs had higher CMO and TMC, while gastrointestinal and cutaneous AEs were those with higher CMN compared with other classes of AEs in the first months that PD-1 inhibitors were used.

The results of this work also demonstrated there is an effect of AE grading only on CMN and TMC in the period of 4 to 6 months after the PD-1 inhibitor was first used. In this case, the greater the severity of the AE, the greater the CMN and TMC.

## Discussion of the results

This study evaluated CMO, CMN, and TMC in quarterly periods from 0 to 24 months of follow-up of cancer patients who used a PD-1 inhibitor from the SSS perspective and provided real-world evidence of the economic burden of AEs associated with this class of drugs available in Brazil since 2016. The real-world data used in this study are similar to previous studies based on medical records with a majority population over 65 years of age, predominantly male patients, and the occurrence of grade 3 AEs in approximately 10% of patients using an immunological checkpoint inhibitor [36, 37]. Furthermore, AEs were more frequent in the first six months after beginning treatment with the PD-1 inhibitor and, in general, they occurred in all tumor types, reinforcing the findings of the previous literature, which showed that AEs are linked more to the drug and patient than the tumor type [16, 18, 20]. Moreover, the anatomical sites most affected by AEs were the gastrointestinal tract and the skin, corroborating the previous literature [16, 17, 36].

The analysis of the economic burden of toxicity showed higher CMO than CMN in all the periods analyzed. In general, for every BRL 100 on average invested in the TMC, BRL 95 are used to monitor the occurrence of the AE and only BRL 5 to manage an identified AE. Considering only the quarterly mean cost with the PD-1 inhibitor drug (BRL 146,793 ± 12,488, ICMS 18%, [46]), the mean TMC represented only 3% of the total expenditure on the drug per patient in the 0 to 3 months follow-up period. In the other follow-up periods, the contribution of toxicity to the economic burden per patient was reduced to 2%. These data corroborate the previous literature. A retrospective analysis conducted by Moura et al. (2021) [36] that considered the total cost of the journeys of melanoma patients showed that drug costs represented almost half of the total cost, while the economic burden of managing AEs represented only 3% of the mean total expenditure per patient.

The clinical benefit of PD-1 inhibitors is recognized globally, although there is a high risk of catastrophic expenditures to access these high-priority cancer drugs [45]. In this respect, for a country like Brazil, where the exclusive cost of treatment with these drugs is a challenge, the additional economic burden of 3% for managing AEs needs to be carefully evaluated. Information on the economic burden associated with AEs can support cost-effectiveness modeling of the use of PD-1 inhibitors in different indications of drug use, as well as support strategic decision-making in the SSS with regard to cost predictability and negotiation processes between health service providers and HMO [15].

The result of multiple paired comparisons corroborates the previous literature by showing that cancer patients under the age of 65 tend to have a lower TMC [37], while cancer patients with a higher level of schooling tend to have higher quality care and better overall health outcomes [47], requiring lower CMN. On the other hand, despite previous research showing that women with metastatic melanoma or non-small cell lung cancer are more likely to have AEs [22] and have higher TMC [37] compared to men, this was not confirmed by the present study. The result of paired multiple comparisons for marital status also does not corroborate with previous literature that sustains that undertreatment and lack of social support in unmarried patients can impact treatment adherence and, consequently, in the occurrence and cost of AEs [40].

Cost comparisons with regard to tumor types showed a trend towards higher CMO and TMC for malignant kidney neoplasms and lower CMO and TMC for malignant bladder neoplasms, which has not been described in the literature to the best of our knowledge. The type of cancer treatment also directly impacts the incidence and severity of AEs, including comparisons of different immune checkpoint inhibitors [16, 18–20, 34]. The prior literature was contradictory when drawing comparisons between the incidence of AEs with the use of nivolumab and pembrolizumab, with results of greater tolerability associated with pembrolizumab [18] or a similar toxicity profile between the products [20]. Data from this study on the economic burden of toxicity associated with the use of PD-1 inhibitors did not translate into differences in CMN between the three drugs in question. The difference in CMO and TMC between PD-1 inhibitors is directly associated with the difference in the dose regimen that favors cemiplimab and pembrolizumab over nivolumab, with the consumption of fewer health resources to monitor AEs. It is important to emphasize that nivolumab can currently be administered in a fixed dose of 480 mg every four weeks. However, this dosage had yet to be approved in Brazil during the period in which the patients in this study used the PD-1 inhibitor.

The line of treatment of cancer patients using PD-1 inhibitors also affects CMO, CMN, and TMC. Health professionals are aware that there is a higher rate of pneumonitis in patients who received the PD-1 inhibitor in the first line of treatment [20, 23], and this justifies a higher CMO, including requests for a higher number of imaging tests for the early identification of this severe AE. However, as far as is known, there are no reports in the literature of higher CMN and TMC resulting from toxicity associated with the use of PD-1 inhibitors after the failure of previous treatment. A previous study even showed that treatment with a PD-1 inhibitor in second-line treatment in patients with non-small cell lung cancer was associated with lower costs for managing grade 3–4 AEs compared with chemotherapy [37]. However, there is no comparison of cost for managing toxicity between the first and second lines of treatment.

Regarding exposure time, CMO is directly proportional to the time of use of the PD-1 inhibitor since the occurrence of AEs must be monitored during treatment and for six months after its interruption [21]. On the other hand, given that the frequency of AEs tends to be reduced over time, CMN also shows a tendency to follow this same reduction pattern. Finally, although the result was not statistically significant, CMN and TMC tended to be higher for the combined regimen of PD-1 inhibitors, which corroborates the previous literature [35].

As expected, TMC was higher for patients with AE than for patients without toxicity. The greater the severity of the AE, the greater the CMN and TMC, since greater consumption of health resources is required to manage the toxicity. Additionally, AEs that generated consumption of inpatient resources were more costly than the others, confirming what has been shown in other countries: that hospitalization is one of the main cost drivers of TMC [20, 36].

Among the AEs, those that affected the lung had higher CMO and TMC, while AEs in the gastrointestinal tract and skin had higher CMN. In addition to the higher rate of occurrence of pneumonitis with the use of PD-1 inhibitors, this AE has the highest fatality rate [16, 23], requiring constant monitoring, especially in patients with baseline risk factors or who will undergo combined treatment with a PD-1 inhibitor [20]. Furthermore, a previous study conducted on patients with melanoma showed that the average cost per patient for AEs in the gastrointestinal tract varied greatly due to the greater variability of data in the literature, higher incidence of AEs regardless of the therapeutic regimen administered, and the high cost of managing toxicity [35]. On the other hand, this same study did not demonstrate a great economic burden for managing cutaneous AEs associated with the use of PD-1 inhibitors [35], as found in this study.

In general, toxicities similar to classic cytotoxic chemotherapy are more predictable than autoimmune toxicities, as they are usually related to the cumulative dose and reserve in specific organs [20], while the homeostatic imbalance of the regulation of the immune system has a wider spectrum of potential damage. Nevertheless, active surveillance is essential regardless of the AE pathogenesis, since the toxicity can be severe and even fatal in some cancer patients, especially those of an autoimmune nature. Unfortunately, there are no clinically validated biomarkers that allow individualized assessments of the potential risk of toxicity for cancer patients. Some predictors of AEs related to immune checkpoint inhibitor therapy involve the patient's genotype, previous history of autoimmune disease, presence of baseline autoantibodies, specific cytokine levels, changes in the rate of certain circulating immune cells, characteristics of the microbiome, the tumor burden [16], and the type of immune checkpoint inhibitor used in cancer patients [20].

On the other hand, there is no formal contraindication to the use of immune checkpoint inhibitors in patients at increased risk of developing an AE. Even so, there is a consensus in the literature that these patients should be regularly monitored by specialized multidisciplinary teams, preferably using a personalized surveillance strategy [20]. Indeed, a broad and validated strategy for the surveillance of cancer patients can spur more assertive clinical management in using innovative therapies, although they can result in a greater economic burden related to CMO and TMC. A full understanding of the economic burden associated with AEs can aid estimates of the incremental costs associated with incorporating new technologies by separating the costs related to monitoring early AE recognition from those necessary to manage an identified AE. Disease cost studies related to the economic burden of treatment toxicity also aid the choice of drug and support economic modeling for decision-making in the country, especially in Brazil, where the health budget does not meet all the needs of the population.

This study has some limitations related to the real-life database. Some information was not available and, for some medical records, the mean follow-up time did not allow for late AEs to be evaluated. Additionally, despite the health service provider having a relatively complete file of cancer patients, the SSS in Brazil is complex, asymmetric, and fragmented, so that a great deal of information was captured by estimates in secondary databases to which the information is sent. This is optional and/or does not capture the confidential business relationships that exist between the healthcare provider and the HMO. Furthermore, although the results are unprecedented in the country, they were limited to a small population of patients treated in only one city and by a single health service provider, which prevents generalizing the results to other realities in Brazil.

Finally, it is important to note that assessing the economic burden of AEs from PD-1 inhibitors represents only one side of the coin. In order to make a decision on the total disease burden from the payer perspective, future research should tackle the issue of how much of the cost of treating AE can be reduced by the use of PD-1 inhibitors compared with conventional chemotherapy. Future research should examine the economic burden of toxicity for different classes of products that make up the therapeutic arsenal in oncology. Furthermore, as standards of clinical practice within and between countries evolve towards early detection and more optimized management of AEs, it would be important to understand to what extent the economic burden of toxicity affects the total cost per patient and how it is used in the economic modeling of cost-effectiveness.

## Practical and managerial contributions

This study makes three contributions to the literature. First, it provides real-world evidence of the economic burden of AEs associated with the use of PD-1 inhibitors. Second, it shows how the economic burden of AEs contributes to the total cost of using PD-1 inhibitors in Brazil. Although previous studies conducted in the United States have shown little relevance regarding the costs of PD-1 inhibitor toxicity regarding the total expenditure per patient with melanoma [36], there are significant differences in care strategies in a pattern of resource use, drug reimbursement status, and choice of treatment by physicians, as well as disease characteristics of patients from one country to another. This limits the extrapolation of data from one geographical region to another [34]. As far as is known, no similar study has been conducted in developing countries, where the isolated cost of the drug is already prohibitive and precludes increasing access to innovative technologies [11].

Finally, this work also makes methodological contributions by evaluating the economic burden of AEs related to PD-1 inhibitors considering the kinetics of toxicity and categorizing costs into CMO, CMN, and TMC. AEs usually develop weeks to months after treatment with PD-1 inhibitors is begun and vary greatly, depending on the affected organ and type of treatment [16, 20]. The wide variability of AEs in their clinical manifestation, incidence, and onset kinetics justifies the importance of clinical and economic analyses with more restricted follow-up periods. Furthermore, for treatments with a good tolerability profile, knowledge of the contribution of each element that makes up the total cost of AE management can contribute to different solutions regarding the total economic burden of toxicity in the health system.

## Conclusion

PD-1 inhibitors represent a new treatment opportunity for cancer patients with different tumor types. Despite the high levels of efficacy, the tolerability of these drugs should not be neglected, as their safety profile requires new skills from healthcare professionals, due to increasing reports of rare forms of AEs. Additionally, there is no clinical or economic pattern concerning the impact of the toxicity of PD-1 inhibitors on global health systems. Given the real-world evidence provided by this study of the economic burden of toxicity associated with the use of PD-1 inhibitors in SSS and all the potential factors that may affect it, Brazil now has the opportunity to incorporate this information into economic analyses that feed the health decision-making process.

## Supplementary Information

**Additional file 1:** **Tab 1S.** Characterization of the rate of occurrence, grading and classification of treatment-related adverse events (AE) by the anatomical site in quarterly periods from 0 to 24 months of follow-up. **Tab 2S.** Comparison of CMO, CMN and TMC between different socio-demographic factors of cancer patients based on the Kruskal-Wallis and Mann-Whitney test. **Tab. 3S.** Comparison of CMO, CMN and TMC between different tumor types based on the Kruskal-Wallis test. **Tab. 4S.** Comparison of CMO, CMN and TMC between different PD-1 inhibitors based on the Kruskal-Wallis test. **Tab. 5S.** Evaluation of CMO, CMN and TMC according to treatment line and time of exposure to PD-1 inhibitor based on the Kruskal-Wallis and Mann-Whitney test. **Tab. 6S.** Evaluation of CMO, CMN and TMC according to the treatment regimen and the need to use hospital resources based on the Mann-Whitney test. **Tab. 7S.** Evaluation of CMO, CMN and TMC between different classifications of AEs during different follow-up times of anti-PD-1 use based on the Kruskal-Wallis test. **Tab. 8S.** Comparison of CMO, CMN and TMC between different grades of AEs during different times of use of a PD-1 inhibitor based on the Kruskal-Wallis test.

## Data Availability

The Relevant data and material are available in the article. Other support data may be requested from the corresponding author.

## Footnotes

1 ^*^ CTCAE uses a range of grades from 1 to 5. Specific conditions and symptoms may have values or descriptive comment for each level, but the general guideline involves: grade 1, Mild; grade 2, Moderate; grade 3, Severe; grade 4, Life-threatening; grade 5, Death.
