# Supplementary material for: Does it fit in your pocket? economic burden of PD-1 inhibitors' toxicity in the supplementary health system: evidence from Brazil
Source: BMC Health Serv Res. 2023 Jul 21;23:781. doi: 10.1186/s12913-023-09736-6 (PMC10360250; doi:10.1186/s12913-023-09736-6)
Supplement: Supplementary file 1 — Additional file 1: Tab 1S. Characterization of the rate of occurrence, grading and classification of treatment-related adverse events (AE) by the anatomical site in quarterly periods from 0 to 24 months of follow-up. Tab 2S. Comparison of CMO, CMN and TMC between different socio-demographic factors of cancer patients based on the Kruskal-Wallis and Mann-Whitney test. Tab. 3S. Comparison of CMO, CMN and TMC between different tumor types based on the Kruskal-Wallis test. Tab. 4S. Comparison of CMO, CMN and TMC between different PD-1 inhibitors based on the Kruskal-Wallis test. Tab. 5S. Evaluation of CMO, CMN and TMC according to treatment line and time of exposure to PD-1 inhibitor based on the Kruskal-Wallis and Mann-Whitney test. Tab. 6S. Evaluation of CMO, CMN and TMC according to the treatment regimen and the need to use hospital resources based on the Mann-Whitney test. Tab. 7S. Evaluation of CMO, CMN and TMC between different classifications of AEs during different follow-up times of anti-PD-1 use based on the Kruskal-Wallis test. Tab. 8S. Comparison of CMO, CMN and TMC between different grades of AEs during different times of use of a PD-1 inhibitor based on the Kruskal-Wallis test. [file 12913_2023_9736_MOESM1_ESM.docx]

**S1. Source of information**

The SBOC guidelines [21] recommend that all patients receiving PD-1 inhibitors routinely undergo clinical and laboratory examination (blood count, liver, kidney, and thyroid functions) before beginning treatment and at each one or two applications, with intervals of four to six weeks during the first six months following the end of treatment. The frequency of medical appointments and laboratory tests to monitor the occurrence of AEs was based on the treatment dosage of the different PD-1 inhibitors (National Health Surveillance Agency; [3-8, 13]) and real-time drug use. In addition, as imaging tests to assess AEs are performed on demand [24], the real frequency of the use of this resource was validated by information from electronic medical records.

The Brazilian regulation of drug prices is the responsibility of the Chamber of Drug Market Regulation (CMED), and the cost of outpatient drugs was evaluated on the CMED list [43] using the Services and Merchandises Circulation Tax (ICMS) of 18%. The treatment dosage of outpatient drugs followed the recommendation of the health service provider's internal guidelines and the SBOC guidelines [21]. This work also considered the service rate for injectable drug treatments for outpatients, and these costs were assessed by the CBHPM Table*. For persistent grade 2 toxicity, the use of prednisone orally at a dose of 1.0 mg/kg/day for seven days (70 kg patient) was considered. In the case of grade 3 or 4 toxicities, the intravenous use of methylprednisolone 125mg/day (patient of 70Kg) for five days was considered. The treatment dosage considered for the use of infliximab was 5 mg/kg (with the second dose repeated after two weeks) for a 70 kg patient, disregarding the leftover infusion solution, as it cannot be used for further use. This study considered the use of 24 mg of ondansetron before the start of four cycles of chemotherapy plus the PD-1 inhibitor for the symptomatic treatment required by some patients. The use of a 0.9%/500 mL saline solution was considered for the dilution of infliximab and for situations in which cancer patients need intravenous hydration. This analysis did not consider the cost of medications purchased directly by the patient, as the present study deals with the SSS perspective.

The cost related to the use of inpatient resources was assessed by the open data portal of the National Supplementary Health Agency**, which corresponds to the Brazilian regulatory agency linked to the Ministry of Health, which is responsible for the Supplementary Health System. This portal lists the inpatient procedures followed by health service providers to treat SSS beneficiaries. The portal also allows an evaluation of the cost of each inpatient procedure and the total cost of hospitalization for a given ICD in different Brazilian states and different years of competence. This study used the 2019 open database for Minas Gerais State** to assess the median total cost related to different reasons for hospitalization associated with the toxicity of PD-1 inhibitors. Inpatient costs were only accounted for in the total cost calculation in the 0 to 12 and 0 to 24 month intervals because the patients' medical records did not indicate for which treatment cycle the hospital intervention was necessary.

The 2019 hospital costs were not adjusted for the 2020 medical inflation rate. The year 2020 was unusual due to the pandemic and, consequently, many people postponed elective health procedures from the SSS perspective. The hospital medical cost variation was -1.9% in 2020 compared with 2019 (Institute of Supplementary Health Studies, <https://www.iess.org.br/cms/rep/vcmh-ago-2021-v2_pdf.pdf>). Any interpretation of the results must take this limitation into account. The frequency of health resource use of outpatient and inpatient treatments related to PD-1 inhibitors’ toxicity was mapped by the electronic medical records.

**Tab 1S**: Characterization of the rate of occurrence, grading and classification of treatment-related adverse events (AE) by the anatomical site in quarterly periods from 0 to 24 months of follow-up.

| **Patients without AEs**  **N (%)** | **Patients with AEs**  **N (%)** | **Graduation of AEs**  **N (%)** | **Classification of AEs**  **N (%)** |
| --- | --- | --- | --- |
| **0 to 3 months of follow-up (n = 170)** | | | |
| 68 (40.0%) | 102 (60.0%)^*^ | Grade 1 = 60 (58.8%)  Grade 2 = 41 (40.2%)  Grade 3 = 1 (1.0%) | GN: 19 (18.6%)  LG: 6 (5.9%)  SM: 1 (1.0%)  GI: 29 (28.4%)  MB: 12 (11.8%)  LD: 1 (1.0%)  SD: 23 (22.5%)  HD: 1 (1.0%)  ED: 10 (9.8%) |
| **4 to 6 months of follow-up (n = 134)** ^***^ *29 deaths and 7 missing data* | | | |
| 60 (44.7) | 74 (55.3%)^**^ | Grade 1 = 15 (20.2%)  Grade 2 = 48 (64.9%)  Grade 3 = 11 (14.9%) | GN: 16 (21.6%)  LG: 7 (9.4%)  SM: 4 (5.4%)  GI: 22 (29.7%)  MB: 2 (2.7%)  RD: 3 (4.1%)  LD: 4 (5.4%)  SD: 7 (9.5%)  ED: 8 (10.8%)  EYD: 1 (1.4%) |
| **7 to 9 months of follow-up (n = 101) ^***^** 46 deaths and 23 missing data | | | |
| 67 (66.3%) | 34 (33.7%) | Grau 1 = 8 (23.5%)  Grau 2 = 25 (73.5%)  Grau 3 = 1 (3.0%) | GN: 10 (29.4%)  LG: 4 (11.8%)  SM: 4 (11.8%)  GI: 5 (14.7%)  MB: 3 (8.8%)  SD: 5 (14.7%)  ED: 3 (8.8%) |
| **10 to 12 months of follow-up (n = 82)** ^***^ *56 deaths and 32 missing data* | | | |
| 62 (75.6%) | 20 (24.4%) | Grade 1 = 6 (30.0%)  Grade 2 = 13 (65.0%)  Grade 3 = 1 (5.0%) | GN: 3 (15.0%)  SM: 1 (5.0%)  GI: 2 (10.0%)  RD: 6 (30.0%)  SD: 6 (30.0%)  ED: 2 (10.0%) |
| **13 to 15 months of follow-up (n = 65) ^***^** 61 deaths and 44 missing data | | | |
| 55 (84.6%) | 10 (15.4%) | Grade 1 = 1 (10.0%)  Grade 2 = 7 (70.0%)  Grade 3 = 2 (20.0%) | GN: 6 (60.0%)  MB: 1 (10.0%)  RD: 1 (10.0%)  LD: 1 (1.0%)  SD: 1 (10.0%) |
| **16 to 18 months of follow-up (n = 47)** ^***^ 64 deaths and 59 missing data | | | |
| 42 (89.4%) | 5 (10.6%) | Grade 1 = 2 (40.0%)  Grade 2 = 2 (40.0%)  Grade 3 = 1 (20.0%) | GN: 2 (40.0%)  MB: 1 (20.0%)  SD: 1 (20.0%)  ED: 1 (20.0%) |
| **19 to 21 months of follow-up (n = 32)** ^***^ 65 deaths and 73 missing data | | | |
| 30 (93.8%) | 2 (6.2%) | Grade 1 = 0 (0%)  Grade 2 = 2 (100%)  Grade 3 = 0 (0%) | GN: 1 (50.0%)  MB: 1 (50.0%) |
| **22 to 24 months of follow-up (n = 21) ^***^** 65 deaths and 84 missing data | | | |
| 21 (100%) | 0 (0%) | NA | NA |

* 51.9% of patients had two different AEs in the same period. The classification included only the highest grade AE or the first occurrence. **27.0% of patients had two different AEs in the same period. The classification included only the highest grade AE or the first occurrence. ^***^Patients with death registered in the period and with insufficient data in the medical record were excluded from the analysis. AE: treatment-related adverse event; GN: general disorders; LG: lung disorders; SM: skeletal muscle disorders; GI: gastrointestinal disorders; MB: metabolic disorder; RD: renal disorder; LD: liver disorder; SD: skin disorder; IR: infusion-related disorder; HD: hematologic disorder; ED: endocrinological disorder; EYD: eye disorder; NA: Information not available.

**Tab 2S**: Comparison of CMO, CMN and TMC between different socio-demographic factors of cancer patients based on the Kruskal-Wallis and Mann-Whitney test.

| Socio-demographic factors | Subgroups | 0 to 12 Months  mean ± SD | | | 0 to 24 Months  mean ± SD | | |
| --- | --- | --- | --- | --- | --- | --- | --- |
|  |  | CMO (BRL) | CMN (BRL) | TMC (BRL) | CMO (BRL) | CMN (BRL) | TMC (BRL) |
| Age | < 65 years old | 8,291 ± 3,565* | 1,605 ± 5,546 | 9,896 ± 6,396 | 10,042 ± 6,686* | 1,619 ± 5,544 | 11,661 ± 8,251* |
|  | ≥ 65 years old | 9,722 ± 3,910* | 1,044 ± 3,502 | 10,765 ± 4,997 | 12,785 ± 7,442* | 1,073 ± 3,503 | 13,858 ± 7,924* |
| Marital status | Married | 9,164 ± 3,670 | 1,029 ± 3,693 | 10,194 ± 4,995 | 11,794 ± 7,307 | 1,056 ± 3,695 | 12,850 ± 7,881 |
|  | Separated | 9,682 ± 4,527 | 2,809 ± 8,962 | 12,490 ± 8,742 | 13,850 ± 8,843 | 2,823 ± 8,959 | 16,673 ± 10,782 |
|  | Widowed | 10,518 ± 3,882 | 435 ± 364 | 10,953 ± 3,855 | 14,159 ± 7,088 | 461 ± 386 | 14,620 ± 6,986 |
|  | Single | 9,013 ± 4,301 | 1,448 ± 2,456 | 10,461 ± 5,488 | 10,449 ± 6,348 | 1,472 ± 2,442 | 11,921 ±7,234 |
| School level | No instruction | 7,929 ± 3,467 | 408 ± 255 | 8,337 ± 3,437 | 10,673 ± 8,175 | 408 ± 255 | 11,081 ± 8,091 |
|  | Basic education | 9,238 ± 3,825 | 1,133 ± 2,038 | 10,371 ± 4,572 | 11,802 ± 7,086 | 1,165 ± 2,033 | 12,967 ± 7,404 |
|  | High school | 9,864 ± 3,991 | 1,085 ± 4,851* | 10,949 ± 5,984 | 13,529 ± 8,082 | 1,106 ± 4,856* | 14,635 ± 8,963 |
|  | University education | 9,062 ± 3,819 | 1,410 ± 4,580* | 10,471 ± 5,512 | 11,018 ± 6,611 | 1,436 ± 4,575* | 12,454 ± 7,548 |
| Gender | Male | 9,285 ± 3,765 | 1,080 ± 3,698 | 10,365 ± 5,038 | 12,295 ± 7,636 | 1,111 ± 3,698 | 13,405 ± 8,153 |
|  | Female | 9,407 ± 4,077 | 1,435 ± 4,974 | 10,842 ± 6,128 | 11,495 ± 6,700 | 1,448 ± 4,972 | 12,943 ± 7,909 |

AE: adverse event; CMO: direct medical cost for monitoring the occurrence of AE; CMN: direct medical cost for managing an identified AE, TMC: total direct medical cost for managing AEs. The mean difference among groups is statistically significant at the 0.05 level (*p < 0.05).

The age of the cancer patient has an impact on CMO in the period of 0 to 12 months (U = 2,299,500; p < 0.05, Mann-Whitney U), 0 to 24 months (U = 2,261,000; p < 0.05, Mann- Whitney U) and exclusively on TMC in the period of 0 to 24 months (U = 2,307,500; p < 0.05). Patients under 65 years of age have lower CMO and TMC. On the other hand, CMN is impacted by the level of education of cancer patients [X2(3) = 8.995; p < 0.05; X2(3) = 10.079; p < 0.05, Kruskal-Wallis] in the periods of 0 to 12 months and 0 to 24 months, respectively. Patients with complete higher education had significantly higher CMN compared to patients with complete secondary education. The result of paired multiple comparisons for gender and marital status of the cancer patient showed no significant difference in CMO, CMN and TMC in the evaluated periods.

**Tab. 3S**: Comparison of CMO, CMN and TMC between different tumor types based on the Kruskal-Wallis test.

| Period (months) | Cost (BRL) | ICD 10 C34  mean ± SD | ICD 10 C43  mean ± SD | ICD 10 C64  mean ± SD | ICD 10 C67  mean ± SD | Others ICDs  mean ± SD |
| --- | --- | --- | --- | --- | --- | --- |
| 0 to 3 | CMO | 4,079 ± 637* | 4,044 ± 486* | 4,473 ± 467* | 3,235 ± 154* | 3,934 ± 771* |
|  | CMN | 235 ± 211* | 93 ± 171* | 1,452 ± 6,833 | 266 ± 270 | 227 ± 244 |
|  | TMC | 4,314 ± 677* | 4,138 ± 447* | 5,925 ± 6,828* | 3,501 ± 253* | 4,161 ± 843 |
| 4 to 6 | CMO | 2,847 ± 611* | 2,884 ± 524* | 3,290 ± 515* | 2,218 ± 223* | 2,640 ± 882* |
|  | CMN | 228 ± 226 | 121 ± 200 | 1,676 ± 7,378 | 162 ± 218 | 156 ± 214 |
|  | TMC | 3,074 ± 587* | 3,005 ± 558* | 4,966 ± 7,378* | 2,381 ± 256* | 2,796 ± 1,010 |
| 7 to 9 | CMO | 2,597 ± 714 | 3,104 ± 725* | 3,131 ± 646* | 2,134 ± 0* | 2,490 ± 551 |
|  | CMN | 69 ± 115 | 63 ± 105 | 101 ± 139 | 63 ± 153 | 38 ± 92 |
|  | TMC | 2,667 ± 718 | 3,167 ± 784* | 3,232 ± 726* | 2,197 ± 153* | 2,527 ± 613 |
| 10 to 12 | CMO | 2,668 ± 576 | 2,874 ± 528* | 3,087 ± 309* | 1,778 ± 871* | 2,312 ± 436* |
|  | CMN | 44 ± 109 | 49 ± 93 | 79 ± 134 | 38 ± 92 | 68 ± 167 |
|  | TMC | 2,712 ± 543* | 2,924 ± 509* | 3,166 ± 267* | 1,816 ± 894* | 2,380 ± 434* |
| 13 to 15 | CMO | 2,671 ± 670 | 2,776 ± 588* | 3,201 ± 0* | 1,467 ± 1,010* | 2,490 ± 616* |
|  | CMN | 12 ± 52 | 53 ± 115 | 78 ± 153 | 0 | 0 |
|  | TMC | 2,682 ± 672 | 2,829 ± 568* | 3,279 ± 153* | 1,467 ± 1,010* | 2,490 ± 616* |
| 16 to 18 | CMO | 2,813 ± 589 | 2,845 ± 487 | 3,094 ± 225 | 2,134 ± 0 | 2,490 ± 616 |
|  | CMN | 20 ± 68 | 18 ± 82 | 68 ± 109 | 0 | 0 |
|  | TMC | 2,834 ± 607 | 2,863 ± 487 | 3,162 ± 280 | 2,134 ± 0 | 2,490 ± 616 |
| 19 to 21 | CMO | 2,896 ± 521 | 2,768 ± 523 | 2,881 ± 477 | 2,134 ± 0 | 2,401 ± 378 |
|  | CMN | 21 ± 57 | 0 | 45 ± 101 | 0 | 0 |
|  | TMC | 2,918 ± 486 | 2,768 ± 523 | 2,926 ± 392 | 2,134 ± 0 | 2,401 ± 378 |
| 21 to 24 | CMO | - | - | - | - | - |
|  | CMN | - | - | - | - | - |
|  | TMC | - | - | - | - | - |
| 0 to 12 | CMO | 8,570 ± 3,716* | 10,905 ± 3,570* | 10,850 ± 3,805* | 6,485 ± 3,127* | 7,589 ± 3,268* |
|  | CMN | 891 ± 1,731 | 608 ± 1,410* | 3,629 ± 9,472* | 714 ± 1,003 | 383 ± 323 |
|  | TMC | 9,460 ± 3,859* | 11,513 ± 4,048* | 14,478 ± 8,915* | 7,199 ± 3,161* | 7,972 ± 3,302* |
| 0 to 24 | CMO | 10,333 ± 6,638* | 15,547 ± 7,726* | 14,184 ± 7,350* | 7,686 ± 4,926* | 8,774 ± 5,356* |
|  | CMN | 900 ± 1,729 | 647 ± 1,412* | 3,697 ± 9,456* | 714 ± 1,003 | 383 ± 323 |
|  | TMC | 11,232 ± 6,567* | 16,194 ± 7,811* | 17,881 ± 10,208* | 8,400 ± 4,849* | 9,157 ± 5,329* |

AE: adverse event; CMO: direct medical cost for monitoring the occurrence of AE; CMN: direct medical cost for managing an identified AE, TMC: total direct medical cost for managing the AEs; ICD 10 C34: malignant neoplasm of the bronchi and lungs; ICD 10 C43: skin malignant melanoma; ICD 10 C64: kidney malignant neoplasm; ICD 10 C67: malignant bladder neoplasm. The mean difference among groups is statistically significant at the 0.05 level (*p < 0.05)

The results of this work demonstrated that the tumor type impacts CMO [X2(4) = 38.908; p < 0.05; X2(4) = 23,979; p < 0.05; X2(4) = 24,545; p < 0.05, Kruskal-Wallis], CMN [X2(4) = 15.893; p < 0.05; X2(4) = 12.702; p < 0.05; X2(4) = 13.079; p < 0.05, Kruskal-Wallis] and TMC [X2(4) = 31.888; p < 0.05; X2(4) = 26.959; p < 0.05; X2(4) = 27.146; p < 0.05, Kruskal-Wallis] in the periods of 0 to 3 months, 0 to 12 months and 0 to 24 months, respectively. Tumor type also impacts CMO [X2(4) = 22.155; p < 0.05; X2(4) = 20.437; p < 0.05; X2(4) = 20.480; p < 0.05; X2(4) = 16.968; p < 0.05, Kruskal-Wallis] and TMC [X2(4) = 20.165; p < 0.05; X2(4) = 17.315; p < 0.05; X2(4) = 24.117; p < 0.05; X2(4) = 18.866; p < 0.05, Kruskal-Wallis], but not CMN, in the periods of 4 to 6 months, 7 to 9 months, 10 to 12 months and 13 to 15 months, respectively. The result of paired multiple comparisons showed no significant difference in CMO, CMN and TMC in the periods of 16 to 18 months and 19 to 21 months. No analysis was performed in the period of 21 to 24 months because one of the dependent variables was constant, preventing selection for the Kruskal-Wallis test. The ICD 10 C64 (malignant neoplasm of the kidney) is the tumor type with the highest CMO and TMC, while the ICD 10 C67 (malignant bladder neoplasm) had the lowest CMO and TMC compared with the other tumor types.

**Tab. 4S**: Comparison of CMO, CMN and TMC between different PD-1 inhibitors based on the Kruskal-Wallis test

| Period (month) | Cost (BRL) | Nivolumab  mean ± SD | Pembrolizumab  mean ± SD | Cemiplimab  mean ± SD |
| --- | --- | --- | --- | --- |
| 0 to 3 | CMO | 4,418 ± 395* | 3,268 ± 77* | 3,279 ± 0* |
|  | CMN | 474 ± 3,347 | 247 ± 234 | 90 ± 201 |
|  | TMC | 4,891 ± 3,368* | 3,515 ± 230* | 3,369 ± 201* |
| 4 to 6 | CMO | 3,207 ± 435* | 2,257 ± 419* | 1,949 ± 169* |
|  | CMN | 558 ± 3,815 | 224 ± 244 | 45 ± 101 |
|  | TMC | 3,766 ± 3,841* | 2,481 ± 496* | 1,994 ± 157* |
| 7 to 9 | CMO | 3,137 ± 661* | 2,151 ± 323* | 2,134 ± 0 |
|  | CMN | 76 ± 120 | 62 ± 110 | 0 |
|  | TMC | 3,212 ± 708* | 2,213 ± 361* | 2,134 ± 0 |
| 10 to 12 | CMO | 3,092 ± 317* | 2,015 ± 427* | 2,134 ± 0* |
|  | CMN | 49 ± 107 | 64 ± 117 | 0 |
|  | TMC | 3,141 ± 271* | 2,080 ± 460* | 2,134 ± 0* |
| 0 to 12 | CMO | 10,163 ± 4,047* | 7,625 ± 2,650* | 6,082 ± 2,017 |
|  | CMN | 1,417 ± 4,930 | 778 ± 1,319 | 135 ± 302 |
|  | TMC | 11,580 ± 5,949* | 8,403 ± 2,894* | 6,217 ± 1,957 |

AE: adverse event; CMO: direct medical cost for monitoring the occurrence of AE; CMN: direct medical cost for managing an identified AE, TMC: total direct medical cost for managing AEs. The mean difference among groups is statistically significant at the 0.05 level (*p < 0.05)

The type of PD-1 inhibitor used by cancer patients also has an impact on CMO [X2(2) = 148,502; p < 0.05; X2(2) = 78,508; p < 0.05; X2(2) = 55.828; p < 0.05; X2(2) = 61.280; p < 0.05; X2(2) = 17.048; p < 0.05, Kruskal-Wallis] and on TMC [X2(2) = 110.805; p < 0.05; X2(2) = 63,819; p < 0.05; X2(2) = 47.692; p < 0.05; X2(2) = 59.953; p < 0.05; X2(2) = 19.471; p < 0.05, Kruskal-Wallis] but not on CMN in the periods of 0 to 3 months, 4 to 6 months, 7 to 9 months, 10 to 12 months and 0 to 12 months, respectively. No analyses were performed in periods of time longer than twelve months due to the unavailability of data in one of the groups of the independent variable. Overall, this analysis showed that nivolumab has higher CMO and TMC than other PD-1 inhibitors, probably due to the difference in dosage regimen between the drugs. There is no significant difference between pembrolizumab and cemiplimab.

**Tab. 5S:** Evaluation of CMO, CMN and TMC according to treatment line and time of exposure to PD-1 inhibitor based on the Kruskal-Wallis and Mann-Whitney test

| Factor | Subgroups | 0 to 3 Months  mean ± SD | | | 10 to 12 Months  mean ± SD | | |
| --- | --- | --- | --- | --- | --- | --- | --- |
|  |  | CMO (BRL) | CMN (BRL) | TMC (BRL) | CMO (BRL) | CMN (BRL) | TMC (BRL) |
| Treatment line | First line | 3,094 ± 535 | 117 ± 201* | 4,021 ± 536* | 3,001 ± 430* | 14 ± 56 | 3,015 ± 441 |
|  | Second line + | 4,092 ± 642 | 456 ± 3,048* | 4,548 ± 3,133* | 2,659 ± 645* | 63 ± 118 | 2,722 ± 633 |
| Factor | Subgroups | 0 to 12 Months  mean ± SD | | | 0 to 24 Months  mean ± SD | | |
|  |  | CMO (BRL) | CMN (BRL) | TMC (BRL) | CMO (BRL) | CMN (BRL) | TMC (BRL) |
| Treatment line | First line | 10,470 ± 3,931 | 485 ± 796 | 10,955 ± 3,950 | 13,948 ± 7,968 | 508 ± 800 | 14,455 ± 7,884 |
|  | Second line + | 9,091 ± 3,818 | 1,346 ± 4,541 | 10,437 ± 5,676 | 11,631 ± 7,153 | 1,371 ± 4,539 | 13,002 ± 8,091 |
| Treatment time | 0-6 months | 5,459 ± 1,630* | 1,959 ± 6,236 | 7,418 ± 6,601* | 5,459 ± 1,630* | 1,959 ± 6,236 | 7,418 ± 6601* |
|  | 7-12 months | 10,514 ± 2,203* | 917 ± 1,512* | 11,431 ± 3,086* | 10,514 ± 2,203* | 917 ± 1,512* | 11,431 ± 3,086* |
|  | 13-18 months | 12,574 ± 2,150* | 393 ± 600* | 12,968 ± 2,074* | 15,994 ± 3,209* | 459 ± 634 | 16,453 ± 3,101* |
|  | More than 19 months | 13,017 ± 2,002* | 660 ± 1,505* | 13,676 ± 2,770* | 22,918 ± 4,097* | 723 ± 1,534* | 23,641 ± 4,367* |

AE: adverse event; CMO: direct medical cost for monitoring the occurrence of AE; CMN: direct medical cost for managing an identified AE, TMC: total direct medical cost for managing AEs. The mean difference among groups is statistically significant at the 0.05 level (*p < 0.05).

Likewise, the line of treatment of cancer patients also impacts CMO in the period of 10 to 12 months (U = 376,000; p < 0.05, Mann-Whitney U) and on CMN (U = 1.515,000; p < 0, 05, Mann-Whitney U) and TMC (U = 1.512,500; p < 0.05, Mann-Whitney U) in the period of 0 to 3 months. Statistically significant differences were not found across all the periods, including the 0-12 month and 0-24 month periods, but overall, CMN and TMC tend to be higher for patients who underwent palliative systemic treatment before using the PD-1 inhibitor, and CMO tends to be higher for patients who used the PD-1 inhibitor as first-line treatment.

The results of this study demonstrated that the time of exposure to PD-1 inhibitor treatment affects CMO [X2(3) = 128.146; p<0.001; X2(3) = 145.692; p < 0.001, Kruskal-Wallis], CMN [X2(3) = 15.573; p<0.01; X2(3) = 10.405; p < 0.05, Kruskal-Wallis] and TMC [X2(3) = 94.480; p<0.001; X2(3) = 124.808; p < 0.001, Kruskal-Wallis] in the periods of 0 to 12 months and 0 to 24 months, respectively. In general, CMO increases with the time of PD-1 inhibitor use, as monitoring remains throughout the treatment and up to 6 months after the drug is discontinued. On the other hand, CMN shows a downward trend over the period of use of the PD-1 inhibitor, with a higher cost in the first quarter, but with a statistically significant reduction only between the second semester and later periods. TMC, in turn, also shows increasing values with the time of use of the PD-1 inhibitor, mainly driven by CMO values.

**Tab. 6S**: Evaluation of CMO, CMN and TMC according to the treatment regimen and the need to use hospital resources based on the Mann-Whitney test

| socio-demographic factor | Subgruops | 0 to 12 Months  mean ± SD | | | 0 to 24 Months  mean ± SD | | |
| --- | --- | --- | --- | --- | --- | --- | --- |
|  |  | CMO (BRL) | CMN (BRL) | TMC (BRL) | CMO (BRL) | CMN (BRL) | TMC (BRL) |
| treatment regimen | monotherapy | 9,080 ± 3,890 | 1,354 ± 4,516 | 10,433 ± 5,723 | 11,555 ± 7,265 | 1,378 ± 4,515 | 12,933 ± 8,201 |
|  | Combination | 10,630 ± 3,484 | 381 ± 384 | 11,011 ± 3,374 | 14,523 ± 7,270 | 408 ± 402 | 14,931 ± 7,109 |
| Use of hospital resource | Yes | 9,320 ± 3,843 | 849 ± 4,086* | 10,169 ± 5,299 | 12,057 ± 7,322 | 874 ± 4,085* | 12,930 ± 8,001* |
|  | No | 9,402 ± 4,237 | 5,420 ± 2,343* | 14,823 ± 5,119 | 11,660 ± 7,656 | 5,455 ± 2,328* | 17,114 ± 7,951* |

AE: adverse event; CMO: direct medical cost for monitoring the occurrence of AE; CMN: direct medical cost for managing an identified AE, TMC: total direct medical cost for managing AEs. The mean difference among groups is statistically significant at the 0.05 level (*p < 0.05)

Finally, it is important to emphasize that the treatment regimen with the PD-1 inhibitor (alone or in combination) does not affect CMO, CMN and TMC in any of the periods evaluated, despite the tendency for costs to be higher with the combined treatment regimen. Conversely, the use of inpatient resources to deal with PD-1 inhibitor toxicity has an effect on CMN (U = 26,000; p < 0.001; U=26,000; p < 0.001, Mann-Whitney U) and on TMC (U = 474,000; p < 0.05; U = 679,000; p < 0.05, Mann-Whitney U) in the period of 0 to 12 months and 0 to 24 months, respectively. Although only 7.6% of patients treated with a PD-1 inhibitor require hospitalization to manage AEs, the costs of inpatient resources meant that CMN and TMC were higher for cancer patients who used this resource.

The AE classification impacts CMO [X2(8) = 19,691; p < 0.05; X2(9) = 26.836; p < 0.05, Kruskal-Wallis], CMN [X2(8) = 47.730; p < 0.05; X2(9) = 36,487; p < 0.05, Kruskal-Wallis] and TMC [X2(8) = 22.758; p < 0.05; X2(9) = 23.362; p < 0.05, Kruskal-Wallis] in the periods of 0 to 3 months and 4 to 6 months of PD-1 inhibitor use, respectively. Within 7 to 9 months after beginning treatment with the PD-1 inhibitor, the AE classification impacts CMO [X2(10) = 31,352; p < 0.05, Kruskal-Wallis], but not CMN and TMC. The result of multiple paired comparisons showed no significant cost difference between the classes of AEs in the other periods evaluated. In general, it can be noted that the subgroups of patients with pulmonary AEs have higher CMO and TMC, while gastrointestinal and cutaneous AEs were those with higher CMN compared with other classes of AEs in the first months of use of PD-1 inhibitors.

There is an effect of AE grading only on CMN [X2(2) = 25.245; p < 0.05, Kruskal-Wallis] and TMC [X2(2) = 18.290; p < 0.05, Kruskal-Wallis] in the period of 4 to 6 months after the onset of the PD-1 inhibitor. In this case, the greater the severity of the AE, the greater the CMN and TMC. The result of multiple paired comparisons showed no significant cost difference between the different grades of AEs in the other periods evaluated.

**Tab. 7S**: Evaluation of CMO, CMN and TMC between different classifications of AEs during different follow-up times of anti-PD-1 use based on the Kruskal-Wallis test

| Period month | Cost  (BRL) | GD  mean ± SD | LG  mean ± SD | SM  mean ± SD | GI  mean ± SD | MB  mean ± SD | RD  mean ± SD | LD  mean ± SD | SD  mean ± SD | IR  mean ± SD | HD  mean ± SD | ED  mean ± SD | EYD  mean ± SD |
| --- | --- | --- | --- | --- | --- | --- | --- | --- | --- | --- | --- | --- | --- |
| 0 to 3 | CMO | 4,037 ± 481* | 5,683 ± 655* | 4,346 ± 0 | 3,923 ± 522* | 4,108 ± 908 | - | 3,279 ± 0 | 3,975 ± 520* | - | 4,346 ± 0 | 3,871 ± 649 | - |
|  | CMN | 142 ± 112* | 311 ± 134 | 184 ± 0 | 499 ± 144* | 373 ± 137* | - | 427 ± 0 | 1,759 ± 7,532* | - | 203 ± 0 | 293 ± 109 | - |
|  | TMC | 4,179 ± 479* | 5,993 ± 568* | 4,530 ± 0 | 4,422 ± 513* | 4,481 ± 907 | - | 3,707 ± 0 | 5,734 ± 7,627* | - | 4,549 ± 0 | 4,164 ± 693* |  |
| 4 to 6 | CMO | 2,734 ± 547* | 4,304 ± 634* | 2,668 ± 616 | 2,619 ± 544* | 3,201 ± 0 | 3,221 ± 508 | 2,857 ± 688 | 2,896 ± 521* | - | - | 2,762 ± 613* | 3,201 ± 0 |
|  | CMN | 220 ± 70* | 280 ± 179 | 320 ± 162 | 506 ± 161* | 225 ± 0 | 214 ± 0 | 9,373±17,954 | 129 ± 177* | - | - | 232 ± 19* | 0 |
|  | TMC | 2,955 ± 575* | 4,583 ± 548* | 2,988 ± 610 | 3,125 ± 571* | 3,426 ± 0 | 3,431 ± 632 | 12,231±18,199 | 3,025 ± 628 | - | - | 2,994 ± 619* | 3,201 ± 0 |
| 7 to 9 | CMO | 2,934 ± 576* | 4,538 ± 534* | 3,420 ± 1,255 | 2,561 ± 584 | 2,845 ± 616 | - | - | 3,201 ± 0 | - | - | 2,845 ± 616 | - |
|  | CMN | 158 ± 109 | 238 ± 27 | 231 ± 68 | 332 ± 100 | 225 ± 0 | - | - | 135 ± 123 | - | - | 225 ± 0 | - |
|  | TMC | 3,092 ± 551 | 4,776 ± 543 | 3,651 ± 1,319 | 2,893 ± 579 | 3,070 ± 616 | - | - | 3,336 ± 123 | - | - | 3,070 ± 616 | - |
| 10 to 12 | CMO | 2,490 ± 616 | - | 2,134 ± 0 | 2,668 ± 754 | 2,845 ± 551 | - | - | 2,668 ± 584 | - | - | 2,134 ± 0 | - |
|  | CMN | 225 ± 0 | - | 184 ± 0 | 353 ± 105 | 250 ± 61 | - | - | 143 ± 171 | - | - | 225 ± 0 | - |
|  | TMC | 2,715 ± 616 | - | 2,318 ± 0 | 3,021 ± 648 | 3,095 ± 514 | - | - | 2,811 ± 433 | - | - | 2,359 ± 0 | - |
| 13 to 15 | CMO | 2,934 ± 446 | - | - | - | 3,201 ± 0 | 3,790 ± 0 | 2,134 ± 0 | 1,208 ± 0 | - | - | - | - |
|  | CMN | 281 ± 87 | - | - | - | 375 ± 0 | 0 | 335 ± 0 | 225 ± 0 | - | - | - | - |
|  | TMC | 3,215 ± 492 | - | - | - | 3,576 ± 0 | 3,790 ± 0 | 2,469 ± 0 | 1,433 ± 0 | - | - | - | - |
| 16 to18 | CMO | 2,935 ± 377 | - | - | - | 3,201 ± 0 | - | - | 3,201 ± 0 | - | - | 3,201 ± 0 | - |
|  | CMN | 300 ± 106 | - | - | - | 225 ± 0 |  |  | 225 ± 0 | - | - | 225 ± 0 | - |
|  | TMC | 3,234 ± 272 | - | - | - | 3,426 ± 0 | - | - | 3,426 ± 0 | - | - | 3,426 ± 0 | - |
| 19 to 21 | CMO | 2,134 ± 0 | - | - | - | 2,134 ± 0 | - | - | - | - | - | - | - |
|  | CMN | 225 ± 0 | - | - | - | 150 ± 0 | - | - | - | - | - | - | - |
|  | TMC | 2,359 ± 0 | - | - | - | 2,284 ± 0 | - | - | - | - | - | - | - |
| 22 to 24 | CMO | - | - | - | - | - | - | - | - | - | - | - | - |
|  | CMN | - | - | - | - | - | - | - | - | - | - | - | - |
|  | TMC | - | - | - | - | - | - | - | - | - | - | - | - |

AE: adverse event; CMO: direct medical cost for monitoring the occurrence of AE; CMN: direct medical cost for managing an identified AE, TMC: total direct medical cost for managing AEs, GN: General disorders; LG: lung disorders; SM: skeletal muscle disorders; GI: gastrointestinal disorders; MB: metabolic disorder; RD: renal disorder; LD: liver disorder; SD: skin disorder; IR: infusion-related disorder; HD: hematologic disorder; ED: endocrinological disorder; EYD: eye disorder. The mean difference among groups is statistically significant at the 0.05 level (*p < 0.05)

**Tab. 8S**: Comparison of CMO, CMN and TMC between different grades of AEs during different times of use of a PD-1 inhibitor based on the Kruskal-Wallis test

| Period (month) | Cost (BRL) | Grade 1  mean ± SD | Grade 2  mean ± SD | Grade 3  mean ± SD |
| --- | --- | --- | --- | --- |
| 0 to 3 | CMO | 3,991 ± 518 | 4,199 ± 914 | 4,346 ± 0 |
|  | CMN | 317 ± 202 | 302 ± 202 | 36,303 ± 0 |
|  | TMC | 4,308 ± 529 | 4,501 ± 910 | 40,649 ± 0 |
| 4 to 6 | CMO | 2,917 ± 778 | 2,828 ± 758 | 3,250 ± 162 |
|  | CMN | 175 ± 147* | 310 ± 176* | 3,795 ± 10,782* |
|  | TMC | 3,091 ± 794* | 3,137 ± 726* | 7,045 ± 10,767* |
| 7 to 9 | CMO | 2,934 ± 494 | 3,138 ± 821 | 5,144 ± 0 |
|  | CMN | 197 ± 80 | 209 ± 112 | 329 ± 0 |
|  | TMC | 3,131 ± 483 | 3,347 ± 814 | 5,473 ± 0 |
| 10 to 12 | CMO | 3,023 ± 436 | 2,462 ± 513 | 2,134 ± 0 |
|  | CMN | 113 ± 123 | 253 ± 69 | 409 ± 0 |
|  | TMC | 3,136 ± 396 | 2,715 ± 507 | 2,543 ± 0 |
| 13 to 15 | CMO | 3,790 ± 0 | 2,688 ± 769 | 2,668 ± 754 |
|  | CMN | 0 | 273 ± 82 | 355 ± 28 |
|  | TMC | 3,790 ± 0 | 2,960 ± 810 | 3,023 ± 783 |
| 16 to 18 | CMO | 3,201 ± 0 | 2,935 ± 377 | 3,201 ± 0 |
|  | CMN | 225 ± 0 | 300 ± 106 | 225 ± 0 |
|  | TMC | 3,426 ± 0 | 3,234 ±272 | 3,426 ± 0 |
| 19 to 21 | CMO | - | 2,134 ± 0 | - |
|  | CMN | - | 188 ± 53 | - |
|  | TMC | - | 2,322 ± 53 | - |
| 22 to 24 | CMO | - | - | - |
|  | CMN | - | - | - |
|  | TMC | - | - | - |

AE: adverse event; CMO: direct medical cost for monitoring the occurrence of AE; CMN: direct medical cost for managing an identified AE, TMC: total direct medical cost for managing AEs. The mean difference among groups is statistically significant at the 0.05 level (*p < 0.05)

*Brazilian Hierarchical Classification of Medical Procedures (CBHPM). Information CBHPM 2020-2021. http://www.sbp.org.br/comunicado-cbhpm-2020-2021/. Access 30.06.2021.

**National Health Surveillance Agency. Brazilian open data portal. Hospital Procedures by State. https://dados.gov.br/dataset/procedimentos-hospitalares-por-uf. Access 15.08.2021.

National Health Surveillance Agency. Brazilian open data portal. Hospital Procedures by State. http://ftp.dadosabertos.ans.gov.br/FTP/PDA/TISS/HOSPITALAR/2019/. Access 15.08.2021.
